# Determinants of Intention to Use Artificial Intelligence-Based Diagnosis Support System Among Prospective Physicians

**DOI:** 10.3389/fpubh.2021.755644

**Published:** 2021-11-26

**Authors:** Anh Quynh Tran, Long Hoang Nguyen, Hao Si Anh Nguyen, Cuong Tat Nguyen, Linh Gia Vu, Melvyn Zhang, Thuc Minh Thi Vu, Son Hoang Nguyen, Bach Xuan Tran, Carl A. Latkin, Roger C. M. Ho, Cyrus S. H. Ho

**Affiliations:** ^1^Institute for Preventive Medicine and Public Health, Hanoi Medical University, Hanoi, Vietnam; ^2^Department of Global Public Health, Karolinska Institutet, Stockholm, Sweden; ^3^Institute of Health Economics and Technology, Hanoi, Vietnam; ^4^Institute for Global Health Innovations, Duy Tan University, Da Nang, Vietnam; ^5^Faculty of Medicine, Duy Tan University, Da Nang, Vietnam; ^6^National Addictions Management Service (NAMS), Institute of Mental Health, Singapore, Singapore; ^7^Center of Excellence in Evidence-Based Medicine, Nguyen Tat Thanh University, Ho Chi Minh City, Vietnam; ^8^Bloomberg School of Public Health, Johns Hopkins University, Baltimore, MD, United States; ^9^Department of Psychological Medicine, Yong Loo Lin School of Medicine, National University of Singapore, Singapore, Singapore; ^10^Institute for Health Innovation and Technology (iHealthtech), National University of Singapore, Singapore, Singapore

**Keywords:** artificial intelligence, diagnosis, theoretical model, intention, medical students

## Abstract

**Background:** This study aimed to develop a theoretical model to explore the behavioral intentions of medical students to adopt an AI-based Diagnosis Support System.

**Methods:** This online cross-sectional survey used the unified theory of user acceptance of technology (UTAUT) to examine the intentions to use an AI-based Diagnosis Support System in 211 undergraduate medical students in Vietnam. Partial least squares (PLS) structural equational modeling was employed to assess the relationship between latent constructs.

**Results:** Effort expectancy (β = 0.201, *p* < 0.05) and social influence (β = 0.574, *p* < 0.05) were positively associated with initial trust, while no association was found between performance expectancy and initial trust (*p* > 0.05). Only social influence (β = 0.527, *p* < 0.05) was positively related to the behavioral intention.

**Conclusions:** This study highlights positive behavioral intentions in using an AI-based diagnosis support system among prospective Vietnamese physicians, as well as the effect of social influence on this choice. The development of AI-based competent curricula should be considered when reforming medical education in Vietnam.

## Introduction

Artificial intelligence (AI) was first introduced some years ago, but in recent years, there has been increasing exploration of the utility and cost-saving of such technology (1, 2). AI brings about great potential in changing existing healthcare practice, from prevention, screening, diagnosis, treatment, and care (2, 3). AI could tap onto data from existing medical records; or even data from smartphones that individuals possess, and data from the applications that individuals use, including their social media posts (3, 4). By using large datasets and employ advanced techniques such as machine learning and deep learning approaches, AI informs more precise predictions of behavioral patterns and understanding of existent medical conditions (3). These benefits would facilitate the clinical decision process, improve the efficacy and accuracy of diagnosis, and diminish physician's workload. Evidence on the utility of AI in healthcare has been widely recorded from dentistry (5), primary care (6), radiology (7), ophthalmology (8) or pathology (9). AI has been recommended for inclusion in routine workflow processes (7). It is thus evident from these studies that the use of AI has been explored in various domains, and it is a promising technology for healthcare.

However, although many reports show the promising role of AI, the usage of AI is still in the early stage. Recent studies indicated low rates of physicians who were familiar or had chances to adopt AI in their clinical practices, even in technologically advanced nations such as 5.9% in South Korea (10) or 23% in the United States (11). Many technological, social, organizational, and individual challenges to apply AI principles in healthcare facilities have been discussed thoroughly in literature (2, 12–15). Nonetheless, the most important factor was physician's attitudes and perceptions toward AI, which can decide whether they would want to integrate AI in their practice or not (13, 14, 16). In healthcare, when the clinical decision is closely related to the patient's lives, health professionals are more likely to be cautious to use new technology in treatment and care; thus, it is not easy for them to trust and use a new product to support their practice.

Health systems can actively involve in the roles of AI adopters and innovators. Therefore, given the rapid expansion of AI applications in healthcare, it is crucial for future health workforces to prepare their capacities, as well as positive perceptions and attitudes to participate in the development of these novel tools. Prior studies indicated some controversial results about the attitudes and intentions to use AI in healthcare practices among medical students. For example, a study in the United States revealed that although the majority of radiology students had a belief in the future role of AI, they felt less interest in the applications of AI in the radiology field (17). Another study in the United Kingdom showed that 49% of medical students were more likely to apply for a radiology career due to AI (18). Understanding determinants of their behavioral intention to use and adopt AI in healthcare delivery is thus necessary for developing medical education curriculum to facilitate AI competence.

In Vietnam, it was been reported in 2019 that more organizations (both healthcare and non-healthcare related) have started developing AI technologies, and utilizing such technologies (19). In 2019, the Vietnam Ministry of Health has issued Decision No. 4888/QD-BYT about the applications and development of smart health care during 2019–2025, which underlines the importance of digital health and strategies to integrate digital health, including AI, into routine health service delivery (20). To date, there remains limited evaluation of AI amongst Vietnamese healthcare services. From our knowledge, there has been only prior publication, that of Vuong et al. (21) that presents a framework seeking to evaluate the AI readiness of the Vietnamese healthcare sector. The authors reported that the implementation of AI in healthcare in Vietnam is limited by several factors, such as the lack of funding; the necessary information infrastructure; and most importantly, the lack of understanding and misunderstanding of AI. Whilst the previous article by Vuong et al. (21) has provided some insights into the challenges with AI implementation and utilization, the review focused on issues at a macro-level, and has not evaluated the perspectives of individual healthcare professionals. For there to be a high uptake rate of AI on the ground, there needs to be an understanding of existing attitudes, preferences, and perspectives of future physicians.

In healthcare, various theories have been used to understand comprehensive facilitating factors in the individual's adoption and acceptance of a novel technology. For instance, several theories included the theory of planned behavior, the theory of diffusion of innovations, the technology acceptance model, or the unified theory of user acceptance of technology (UTAUT). Of which, UTAUT has been recognized as one of the most common theories to examine the adoption behavior of one individual (22–25). UTAUT was developed based on other dominant behavioral theories. Venkatesh et al. showed a higher explanatory level of UTAUT compared to other theories in exploring the information technology adaptation, with 70% of the variance for behavioral intentions and 50% of the variance for actual use (26, 27). A previous study in Chinese physicians showed that initial trust and performance expectancy were significant predictors for the AI adoption intentions (28). This study aimed to use UTAUT to explore the behavioral intentions of medical students to adopt an AI-based Diagnosis Support System. Understanding medical student's attitudes and perspectives would help to resolve potential barriers in adoption at the ground level, and such a survey would also help guide AI policy formulation at different levels.

## Materials and Methods

In this section, we presented literature review and conceptual framework of this study. Moreover, study design, data collection method, and statistical analysis were described.

### Literature Review and Conceptual Framework

UTAUT has been used widely in the literature to examine the behaviors of an individual in adopting the technology. UTAUT explains individual's behaviors *via* four constructs: (1) performance expectancy, (2) effort expectancy; (3) social influence, and (4) facilitating conditions (26). Because AI-based Diagnosis Support System has not been implemented in entire Vietnam, we supposed that there was very difficult for medical students to have a chance to use AI systems during their clerkship or when they studied in the medical university. Therefore, we used UTAUT to explore the behavioral intention, which was defined as the willingness of medical students to use this system in the future if they had an opportunity. The behavioral intention was a significant predictor of actual use; thus, it is valid to determine the factors associated with the behavioral intention of AI use, which would partly reflect the AI practice in the future (13).

Firstly, three main constructs of the UTAUT model (i.e., performance expectancy, effort expectancy, and social influence) were included. The performance expectancy refers to “*the degree to which a person believes that using a particular system would enhance his or her job performance*,” while the effort expectancy is defined as “*the degree of ease associated with the use of the system*,” and the social influence refers to “*the degree to which an individual perceives that important others believe he or she should use the new system*” (26). All of them have been revealed to have positive associations with behavioral intentions in different studies regarding IT adoption (26). Performance expectancy is found to be related to effort expectancy because it is supposed that people were more likely to perceive that one technology is useful if they ease using this technology (29). In literature, previous studies showed that medical students believed that AI would help to enhance the performance of practices and AI would be integrated deeply in healthcare, from administrative works to clinical routine (30–33). Indeed, medical students are considered to have high AI literacy than current health professionals. A survey in the United States indicated that medical students were more likely to have basic knowledge about AI and prefer to use AI in patient care when comparing to their faculties (31). Another survey in the United Kingdom found that medical students who were taught about AI were more likely to adopt AI in their practices (18). Social influence may also affect the intention to use AI in healthcare. Prior research in both the general public and health professionals recommended that medical students should learn and practice AI during their studies (31, 34–36). Experts shared that future physicians should have a good understanding and can transforming AI from potential threats to become helpful assistants (37).

*Via* literature review, we also decided to develop the model with three additional constructs: task complexity, personal innovativeness in IT, and technology characteristics. Task complexity is the level of difficulty for completing an assigned task (38); hence, technology can have different roles in different tasks. Health professionals in their daily practices will face a variety of tasks, from simple to complex tasks. If they perceived that their tasks are difficult, they are more likely to accept the support from the AI system to increase their performance (i.e., performance expectancy). A study in Canada showed that medical students perceived the usefulness of AI in providing diagnosis, prognosis, building personalized medication, and performing robotic surgery, which indicated the promising roles of AI in addressing task complexity (33). Meanwhile, personal innovativeness in information technology (IT) means that one person is willing to try an innovation (particularly in IT) (39), while technology characteristics refer to the system, interface, etc. which allow users to use the technology for completing their tasks (40). Prior studies showed the potential relationships between these two constructs with effort expectancy (41, 42). Overall, we attempted to examine the association between task complexity and performance expectancy; and between personal innovativeness in IT and technology characteristics with effort expectancy.

Along with these three constructs, we added perceived substitution crisis and initial trust constructs aiming to examine the facilitating conditions to behavioral intentions. Perceived substitution crisis was served as a potential barrier for medical students to adopt technology in their future practice. Several obstacles such as the likelihood of being replaced by AI, being dependent on AI, being unemployed due to AI, and decreasing diagnosis capacity due to AI would greatly affect the benefits of physicians. Previous research found that 17% of German medical students agreed that AI could replace health professionals (30), and 49% of English medical students stated that they did not prefer the radiology field because of AI (18). Therefore, the perceived substitution crisis was suggested to be included when examining the intention to use AI among health professionals (2, 12–15).

For the initial trust, Mcknight et al. defined trust in the field of technology as “*beliefs about a technology's capability rather than its will or its motives”* (43). Trust is an important determinant of technology acceptance and adoption (44–47). Physicians are more likely to be cautious when adopting new technology in patients to prevent any potential harm; thus, trusting can help to reduce any suspicions and facilitate the use of the AI system among physicians. In a previous study, lack of trust in AI was the main contributor to the negative attitude among Chinese people toward the application of AI in healthcare (34). Another study in Canada found that medical students did not believe AI could deliver personalized and empathetic care (33). Thus, we hypothesized that trust would be positively associated with the behavioral intention to use AI systems in medical students. Given the matter that in Vietnam's medical education curriculum, none of course about AI was tough, we supposed that our medical students did not have any previous experience with AI and AI-based Diagnosis Support System. Thus, among different stages in trust formation, we concentrated on the initial stage, i.e., initial trust, which reflected how people trust in a technology that they have no experience.

**Figure 1:**
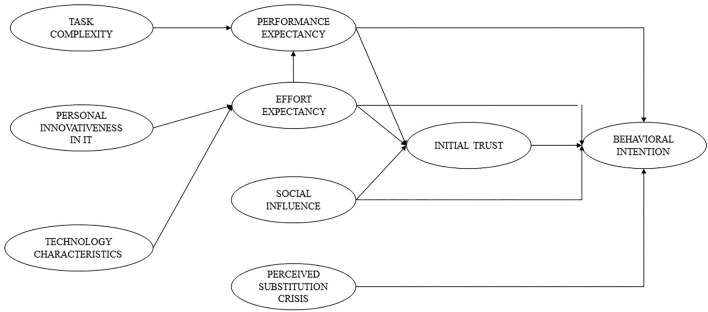
A theoretical model to explore trusts and intentions to use AI-based diagnosis support system.

Additionally, to identify the relationship between initial trust and behavioral intention, we developed a trust-based theoretical model to explore the trust of medical students in a novel technology as an AI-based Diagnosis Support System. We estimated the associations between performance expectancy, effort expectancy, and social influence with initial trust. Previous studies indicated that performance expectancy and effort expectancy were two forms of technology-specific expectations as discussed above, which are believed to result in trust formation (48). Social influence was also found to be an associated factor with trust in other settings. Prior research revealed that those without any experience with technology were more likely to be dependent on the opinions of their important people, which in turn formulated their trust (48–50). The final conceptual framework used in this study is illustrated in Figure 1.

### Study Design and Data Collection

Data of this study was obtained from an Internet survey from December 2019 to February 2020. This online survey was designed by using an online platform called Survey Monkey (https://www.surveymonkey.com/), which is a highly secure online platform. This survey was sent to medical students at medical university in Vietnam, with the inclusion criteria as follow: (1) aged 18 years or above; (2) currently studying undergraduate medical doctor programs in a medical university in Vietnam; (3) having a valid online account (such as email or social network sites) to help to recruit other medical students. We used the snowball sampling technique to recruit participants. First, we sent out the survey to a core group with twenty medical students who were from different medical universities. After they completed the survey, they were asked to invite other medical students in their networks to do the survey. The recruitment chain stopped when no one was invited or completed the survey within 7 days. A total of 223 medical students from different provinces (Hanoi, Ho Chi Minh city, and other provinces) were enrolled in the study. We obtained their electronic informed consent before doing the survey. After excluding invalid responses, data of 211 (completion rate 94.6%) medical students were used for analysis.

### Variables and Questionnaire

In this study, we developed a structured questionnaire with two parts: the demographic characteristics section (including age, gender, living area, specialty, and location), and 26 items that reflected the 9 latent constructs for our theoretical models. These items were about performance expectancy (PE), effort expectancy (EE), social influence (SI), task complexity (TC), personal innovativeness in IT (PI), technology characteristics (TECH), perceived substitution crisis (PC), initial trust (IT), and behavioral intention. These items were selected based on a literature review (26, 28, 41, 42, 48). Participants were asked to respond using a 5-point Likert scale ranging from “strongly disagree” (1), “disagree” (2), “somewhat agree” (3), “agree” (4) to “strongly agree” (5). The proposed constructs and profiles are shown in the Supplementary Material.

### Data Analysis

Stata software version 15.0 was used to analyze the data. Properties of measurement were evaluated. Internal consistency reliability was assessed by using Cronbach's alpha. Good internal consistency was defined as a Cronbach's alpha ≥0.7. Validity was examined, including convergent, discriminant, and construct validities. Convergent validity was assessed *via* two criteria: factor loading >0.70 and average variance extracted of each construct ≥0.5 (41). Regarding discriminant validity, we computed the variance inflation factor (VIF) to examine the multicollinearity of each construct. Construct with VIF value >10 indicated that it was not appropriate as a component of regression analysis. The square root of AVE per construct was also computed, and good discriminant validity was achieved when the square root of AVE of a construct was higher than its correlations with other constructs. Given that a sample size of 211 medical students might not be sufficient for the structural equation modeling (SEM) method, we employed partial least squares (PLS) SEM, which is a 2nd-generation SEM, to assess the relationship between latent constructs. We considered a statistical significance when the *p* < 0.05.

## Results

Table 1 depicts the demographic characteristics of our sample. The mean age of selected medical students was 20.6 years old (SD = 1.5). The majority of them were female at 73.5%, lived in urban areas (89.1%), and Ho Chi Minh city (59.7%). Most of the respondents belonged to the general physician program (57.8%).

**Table 1:** Characteristics of respondents (*n* = 211).

**Characteristics**
Age, years, Mean (SD)	20.6 (1.5)
Gender, *n* (%)
Male	55 (26.5)
Female	155 (73.5)
Living area, *n* (%)
Urban	188 (89.1)
Rural	23 (10.9)
Specialty
General physician	122 (57.8)
Odonto-Stomatology	48 (22.7)
Traditional medicine	41 (19.4)
Location
Hanoi	51 (24.2)
Ho Chi Minh city	126 (59.7)
Other provinces	34 (16.1)

Table 2 showed that the initial trust construct had the lowest mean score at 3.0 (SD = 0.9), while TC had the highest mean score at 3.8 (SD = 0.9). Overall, the Cronbach's alpha of each construct ranged from 0.738 to 0.909, suggesting good reliability among constructs. All item loadings of these constructs were above 0.7, and all construct's AVE values were above 0.5, indicating good convergent validity.

**Table 2:** Reliability and validity of the measure (*n* = 211).

**Factor**	**No. of items**	**Factor loading**	**Mean**	**SD**	**Cronbach's alpha**	**AVE**
PE	4	0.847–0.915	3.7	0.8	0.903	0.775
EE	2	0.945–0.953	3.3	0.9	0.89	0.901
SI	4	0.827–0.894	3.4	0.7	0.88	0.736
PI	4	0.771–0.869	3.4	0.7	0.854	0.696
TC	2	0.879–0.901	3.8	0.9	0.738	0.791
TECH	3	0.824–0.916	3.1	0.8	0.846	0.765
PC	4	0.710–0.862	3.1	0.8	0.825	0.646
IT	2	0.957–0.957	3	0.9	0.909	0.916
BI	1	–	3.4	0.9	–	1

*PE, performance expectancy; EE, effort expectancy; SI, social influence; PI, perceived innovativeness in IT; IT, initial trust; TC, task complexity; TECH, technology characteristics; PC, perceived substitution crisis; BI, behavioral intention*.

In Table 3, regarding discriminant validity, the value of the square root of AVE per construct was higher than its correlation coefficient with other constructs. Moreover, the results of VIF analysis showed that all VIF values were below 10, suggesting no multicollinearity existed.

**Table 3:** Correlation of latent variables and square root of AVE of each construct (*n* = 211).

	**PE**	**EE**	**SI**	**PI**	**IT**	**TC**	**TECH**	**PC**	**BI**
PE	0.8803^*^								
EE	0.6936	0.9492^*^							
SI	0.6794	0.6656	0.8579^*^						
PI	0.7408	0.7391	0.7243	0.8343^*^					
IT	0.4937	0.5586	0.6834	0.5427	0.9571^*^				
TC	0.6002	0.5015	0.5763	0.6523	0.3213	0.8894^*^			
TECH	0.5527	0.6099	0.691	0.5801	0.7728	0.3925	0.8746^*^		
PC	0.3873	0.4568	0.523	0.463	0.3192	0.3935	0.4374	0.8037^*^	
BI	0.5458	0.5453	0.6856	0.5755	0.4904	0.4838	0.4686	0.3729	1.000^*^

*PE, performance expectancy; EE, effort expectancy; SI, social influence; PI, perceived innovativeness in IT; IT, initial trust; TC, task complexity; TECH, technology characteristics; PC, perceived substitution crisis; BI, behavioral intention*.

* *Squared root of AVE*.

**Figure 2:**
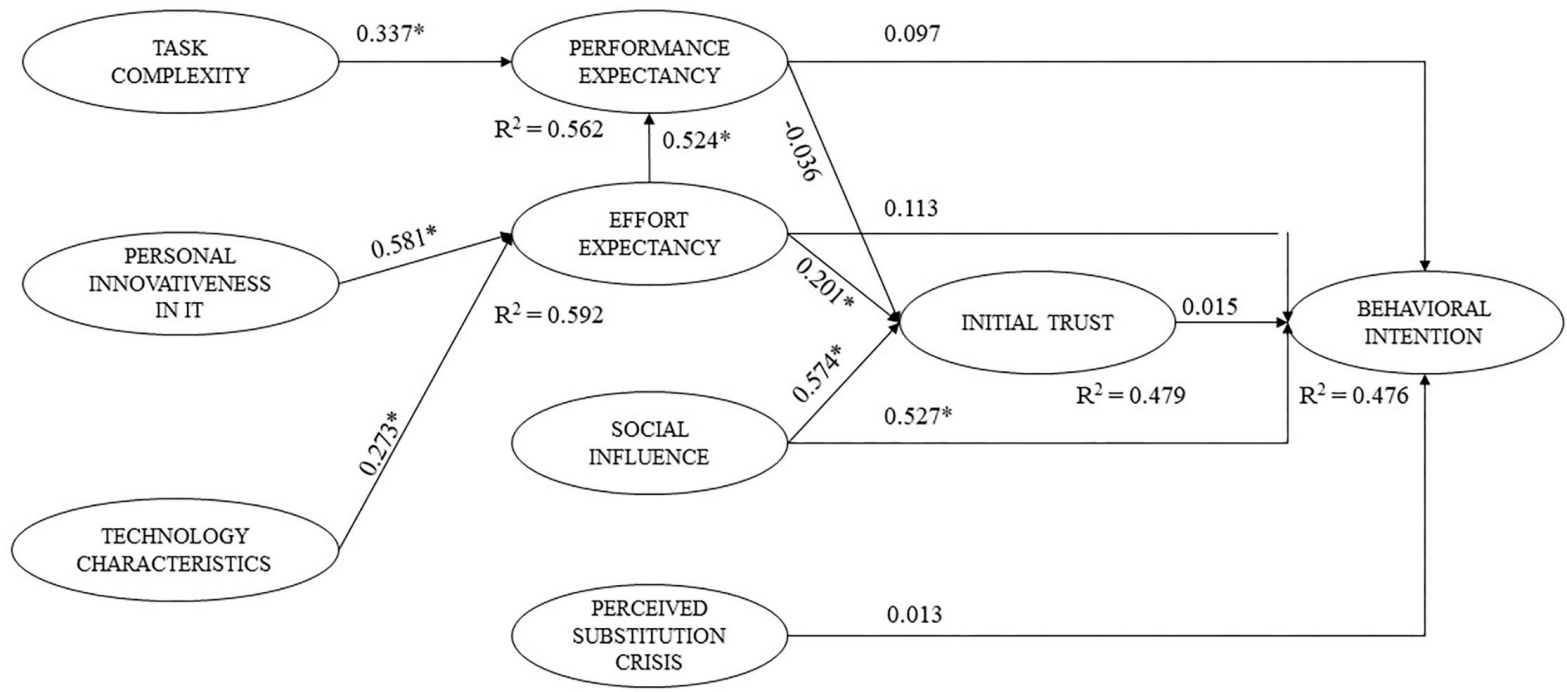
Structural model and standardized path coefficients (*n* = 211). *p < 0.05.

Figure 2 illustrates path coefficients and *p*-values of PLS analysis. Regarding the behavioral intention model, only social influence (β = 0.527, *p* < 0.05) was positively related to the behavioral intention. Meanwhile, other constructs such as performance expectancy, effort expectancy, initial trust, and perceived substitution crisis showed no associations with behavioral intentions to use AI. Overall, the model with five proposed constructs for behavioral intentions, including performance expectancy, effort expectancy, social influence, perceived substitution crisis, and initial trust, explained 47.6% (*R*^2^ = 0.476) of the behavioral intention's variance.

Figure 2 also shows that effort expectancy (β = 0.201, *p* < 0.05) and social influence (β = 0.574, *p* < 0.05) were positively associated with initial trust, while no association was found between performance expectancy and initial trust (*p* > 0.05). The model including performance expectancy, effort expectancy, and social influence explained 47.9% of the variance of initial trust (*R*^2^ = 0.479).

## Discussion

Developing and adopting AI in healthcare are essential due to its great benefits in enhancing healthcare professional's performance and efficiency. Overall, the perceptions of our students about diagnosis-related capacities of AI, effort to use AI, and intention to use AI were positive. It is clear that the role of AI in healthcare delivery has been widely documented, where AI has shown its success in the interpretation of image and examination data, as well as clinical outcomes prediction and management (15, 51). Nonetheless, information about AI and its application in Vietnam has been disseminated in mainstream media but not in university settings. Our results indicated that undergraduate medical students in Vietnam had great confidence in the knowledge of their work characteristics, understanding how AI could assist them to promote diagnosis performance, and desire to use AI when available. However, there were still some gaps between their expectancy and preparation, including awareness of technology characteristics and capacities to use such technology. Equipping the medical students with the basics, as well as the correct understanding and attitudes about the application of AI in medicine, are crucial in the digitalization of the healthcare system. However, currently, the medical training program in Vietnam has not been systematically updated in this area. The AI content has been mainly shared through scientific seminars or short-term courses, without a specific program to develop the AI capabilities.

This lack of pre-paration might also lead to the findings that the majority of our sample somewhat agreed or agreed that AI would replace the position of physicians in healthcare. This result was congruent with findings among medical students worldwide, particularly those in the radiology field (13, 14, 18, 52). Several previous studies found contradict results where the medical students stated that AI could not have a role as an alternative for the physicians in the future (33, 53), particularly in some fields that need a “sense of caring” or “art of caring” such as psychological health or aging care (53–55). Many authors argued that AI should be treated as a virtual assistant rather than being a replacement for physicians in healthcare. However, prospective physicians should acquire fundamental knowledge about mathematics, data science, AI, as well as ethical and legal issues related to AI (56). They should understand the systemic bias behind AI algorithms due to the insufficient data, which might be a great reason for health equity issues when making a clinical decision (57, 58). Moreover, other humanistic aspects such as communication skills, empathy, decision-making, or leadership skills should also be required (53). Acquiring these capacities would enable physicians to take advantage of AI in integrating it into their routine clinical practices. Thus, it is needed to call actions to innovate the medical education programs in the digital area.

Our path analysis showed the dominance of social influence on the intention of using AI for future work among undergraduate medical students, instead of other factors such as performance expectancy or initial trust, which were found in the previous research (28). Although this result is unexpected compared to what we hypothesized, there were several reasons which can be used to explain this phenomenon. First, this study was conducted on undergraduate medical students, whose healthcare delivery experience, as well as perceptions about the diagnosis process, were constrained. Moreover, given that AI has not yet been scaled up in Vietnam and AI-related curriculums for medical students had not yet been developed, we supposed that the majority of our sample had no experience with an AI-based diagnosis support system. This limitation hinders the way medical students perceived their capacities in adopting AI, as well as results in the homogeneity in their competency and trust evaluation. Moreover, because of this lacking experience, it is understandable when undergraduate medical students tended to be heavily dependent on the experiences of senior physicians in their social networks and information they gathered in social media about AI. With the exchange and sharing of practical experiences from those who have used this AI system, students' trust and intention to use the AI system in the future would be improved.

The findings of this study suggested several implications. First, undergraduate medical students should actively find opportunities to update and involve in AI development and adoption to increase their necessary AI knowledge and capacities. Self-learning ability is important to acquire new knowledge in the context where AI curricula at medical schools have not been paid sufficiently. Second, our study suggested the importance of role model approaches for facilitating the use of AI in this group. Opportunities to gain hands-on experience in different teaching hospitals are critical. AI may be useful for diagnosing rare conditions, which are often only seen at large teaching hospitals. Finally, this study underlined the need to integrate AI curriculums in the current medical education, which helped medical students to prepare appropriate capacities in technology adoption. Further studies should be performed to measure the preference and effectiveness of different education strategies to facilitate AI applications in healthcare among health professionals and medical students. Moreover, they should also assess whether training students with AI helps or hinders their diagnostic abilities.

Some limitations should be acknowledged in this study. First, since our study was conducted on medical students who had no experience with AI-based diagnostic support systems, we have not yet assessed whether they would use these systems or not in the future. A longitudinal follow-up study evaluating the rate of use of this system among medical students after graduation is essential to help refine the theoretical model. Second, our research was conducted online and had recruited medical students in entire Vietnam; however, this study may be limited to the group of medical students with Internet access, while other groups of medical students were not accessed. In addition, a small sample size might reduce the statistical power. Other studies on larger sample sizes need to be conducted, which help verify our results in other medical student groups. Third, in addition to constructs included in the theoretical model, the study has not assessed the mediating effects of other factors such as age, gender, and previous training in AI use during university studies, etc., which could affect the relationship among factors in the theoretical model.

## Conclusions

This study highlights positive behavioral intentions in using an AI-based diagnosis support system among prospective Vietnamese physicians, as well as the effect of social influence on this choice. The development of AI-based competent curricula should be considered when reforming medical education in Vietnam.

## Data Availability Statement

The raw data supporting the conclusions of this article will be made available by the authors, without undue reservation.

## Ethics Statement

The studies involving human participants were reviewed and approved by Institutional Review Board of Youth Research Institute. The patients/participants provided their written informed consent to participate in this study.

## Author Contributions

LN: conception, design, acquisition, interpretation of data, drafting the article, and final approval of the version to be published. HN: conception, design, drafting the article, and final approval of the version to be published. AT: design, analysis, interpretation of the data, drafting the article, and final approval of the version to be published. CN: acquisition, analysis, interpretation of data, drafting the article, and final approval of the version to be published. LV and MZ: conception and design of data, revising article, and final approval of the version to be published. CH: conception and design of data, drafting the article, and final approval of the version to be published. BT, CL, and RH: conception, design, interpretation of data, drafting the article, and final approval of the version to be published. All authors contributed to the article and approved the submitted version.

## Funding

This study was funded by NUS iHeathtech Other Operating Expenses (R-722-000-004-731) and NUS Department of Psychological Medicine Other Operating Expenses (R-177-000-003-001).

## Conflict of Interest

The authors declare that the research was conducted in the absence of any commercial or financial relationships that could be construed as a potential conflict of interest.

## Publisher's Note

All claims expressed in this article are solely those of the authors and do not necessarily represent those of their affiliated organizations, or those of the publisher, the editors and the reviewers. Any product that may be evaluated in this article, or claim that may be made by its manufacturer, is not guaranteed or endorsed by the publisher.
