# Supplementary material for: Determinants of Intention to Use Artificial Intelligence-Based Diagnosis Support System Among Prospective Physicians
Source: Front Public Health. 2021 Nov 26;9:755644. doi: 10.3389/fpubh.2021.755644 (PMC8661093; doi:10.3389/fpubh.2021.755644)
Supplement: Supplementary file 1 [file Table_1.DOCX]

**Supplement. Profiles of constructs**

| **Construct** | **Items** | **Strongly disagree** | **Disagree** | **Somewhat agree** | **Agree** | **Strongly agree** |
| --- | --- | --- | --- | --- | --- | --- |
|  |  | **%** | **%** | **%** | **%** | **%** |
| Performance expectancy | 1. Using AI would enable me to accelerate my diagnosis | 3.3 | 3.8 | 26.5 | 51.2 | 15.2 |
|  | 2. Using AI would enhance my work performance | 2.8 | 4.3 | 25.6 | 52.1 | 15.2 |
|  | 3. Using AI would promote my diagnosis level | 3.8 | 6.6 | 34.1 | 45.5 | 10.0 |
|  | 4. Using AI would release my work pressure | 2.8 | 4.7 | 19.9 | 55.5 | 17.1 |
| Effort expectancy | 5. Learning to use AI would be easy to me | 3.8 | 11.9 | 38.9 | 39.8 | 5.7 |
|  | 6. I Think I could operate AI skillfully | 4.3 | 10.4 | 43.6 | 35.1 | 6.6 |
| Social influence | 7. 1 I think many doctors are using AI | 3.8 | 5.2 | 46.5 | 39.8 | 4.7 |
|  | 8. I think my leaders would want me to use AI | 3.3 | 5.2 | 51.2 | 35.6 | 4.7 |
|  | 9. I think my patients would want me to use AI | 4.3 | 6.6 | 55.0 | 28.4 | 5.7 |
|  | 10. I think using AI is an indisputable trend | 3.8 | 5.7 | 33.2 | 49.3 | 8.1 |
| Personal innovativeness in IT | 11. I usually keep an eye on emerging technology products | 4.7 | 6.6 | 35.6 | 42.2 | 10.9 |
|  | 12. I always try out new technology products earlier compared to others | 5.7 | 20.9 | 46.9 | 22.8 | 3.8 |
|  | 13. In general, I am willing to accept new technology things | 3.3 | 2.4 | 30.3 | 51.7 | 12.3 |
|  | 14. If I heard about a new technology product, I would look for ways to operate it | 3.8 | 2.4 | 30.3 | 51.7 | 11.9 |
| Initial trust | 15. I believe AI could provide completely accurate diagnosis assistant service | 9.0 | 21.8 | 43.6 | 21.3 | 4.3 |
|  | 16. I believe AI could provide completely reliable diagnosis assistant service | 7.1 | 19.9 | 41.2 | 27.0 | 4.7 |
| Task characteristic | 17. My misdiagnosis or missed diagnosis would lead to serious results on patients | 5.2 | 0.5 | 24.6 | 35.1 | 34.6 |
|  | 18. I usually need to analyze lots of medical data (medical images data, medical text data, etc.) for diagnosis. | 4.7 | 3.8 | 34.1 | 38.9 | 18.5 |
| Technology characteristics | 19. I think diagnosis accuracy rate of AI would be higher than that of common doctors | 7.6 | 26.1 | 41.2 | 20.9 | 4.3 |
|  | 20. I think diagnosis speed of AI would be faster than that of common doctors | 4.7 | 10.9 | 45.0 | 35.6 | 3.8 |
|  | 21. I think AI have a clear and understandable diagnosis process | 6.2 | 16.1 | 48.3 | 25.1 | 4.3 |
| Perceived substitution crisis | 22. I think that AI would likely to replace doctors in the future | 15.6 | 28.4 | 37.0 | 16.6 | 2.4 |
|  | 23. I think using AI for a long time would make doctors dependent on them | 6.6 | 9.0 | 33.2 | 41.7 | 9.5 |
|  | 24. I think the rise and development of AI would likely to lead unemployment of some doctors | 8.1 | 15.2 | 40.8 | 29.9 | 6.2 |
|  | 25. I think using AI for a long time would decrease doctors’ own diagnosis ability | 4.7 | 11.4 | 40.8 | 35.6 | 7.6 |
| Behavioral Intentions | 26. I make sure I will use AIDSS if I have an opportunity. | 5.2 | 3.3 | 42.2 | 41.2 | 8.1 |
